# Exercise-based interventions for problematic short-video use: a narrative review of current evidence and potential psychological pathways

**DOI:** 10.3389/fpsyg.2026.1865897

**Published:** 2026-09-03

**Authors:** Wenbo Hu, Donghui Tang

**Affiliations:** College of P.E. and Sports, Beijing Normal University, Beijing, China

**Keywords:** exercise, narrative review, physical activity, problematic short-video use, psychological processes, self-control, short-video addiction

## Abstract

Problematic short-video use has become an emerging digital health concern among adolescents and college students. In this review, problematic short-video use is not treated as an independent clinical disorder with formal diagnostic status. Instead, it is conceptualized as an addiction-like pattern of digital-media use characterized by impaired control, psychological dependence, and real-world functional impairment. Using a theory-driven narrative review approach, this article organizes short-video-specific and transferable evidence around four overlapping pathways that may link exercise with problematic short-video use: reward substitution, emotional restoration, control restoration, and real-world reconnection. These pathways are theoretical propositions rather than established mechanisms, and support for them is uneven. Control restoration has the most direct, although still limited, support because it includes one small controlled exercise study; emotional restoration is supported mainly by short-video-specific association studies; and reward substitution and real-world reconnection rely more heavily on indirect or transferable evidence. The overall efficacy of exercise remains uncertain, and practical implications are preliminary because exercise type and dose, target populations, adherence, and implementation conditions have not been established. By distinguishing direct, reverse-direction, contextual, and transferable evidence, this review provides a structured synthesis of the field and identifies priority outcomes and design considerations for future controlled studies.

## 1 Introduction

The widespread adoption of short-video platforms has made short-video viewing a routine part of everyday life. These platforms typically combine brief content, continuous swiping, personalized recommendation, and low-threshold access (Kong and Sang, 2017; Liao, 2024). Adolescents and college students encounter these features in different developmental and educational contexts, which should be considered when findings from the two populations are compared. Reported problems include difficulty controlling use, displacement of daily activities, academic procrastination, and broader functional impairment (Dong et al., 2023; Huang, 2023; Song et al., 2025; Zhao and Lu, 2024). In this review, the phenomenon often described as “short video addiction” is discussed under the broader term problematic short-video use, without treating it as a formally recognized clinical disorder.

Research on problematic short-video use has approached the issue from several directions. Studies focused on problematic-use characteristics emphasize loss of control and functional impairment (Zhao and Lu, 2024); platform-focused work examines personalized recommendation, content compression, and immersive interaction (Liao, 2024); and related psychological research has considered emptiness and compensatory expectations (He, 2024) as well as mobile short-video attachment (Jin and Liu, 2023). Together, these strands help explain why short-video use may become difficult to regulate. Exercise and physical activity, however, have received much less direct study.

Li J. et al. (2025), Li T. et al. (2025), and Li Y. et al. (2025) recently provided a systematic review of problematic short-video use, synthesizing its conceptual status, theoretical and neural mechanisms, negative effects, and measurement tools. That review offers a broad psychological overview of the phenomenon but does not focus on exercise as a candidate intervention or translate the available evidence into intervention-oriented hypotheses. The present review addresses this complementary gap by asking how short-video-specific and transferable evidence can inform the design, outcome selection, and empirical testing of exercise-based interventions.

Exercise warrants attention because it is structured and repeatable and can involve sustained effort, skill development, self-regulatory demands, and, depending on the activity, social participation. These characteristics make it conceptually relevant to motivation and reward, emotion regulation, self-control, and offline connectedness. Current support is uneven: most directly relevant studies are cross-sectional or mediation-based (He et al., 2024; Wang G. et al., 2025; Wu et al., 2026), only one small controlled exercise study is available (Wang K. et al., 2025), and some proposed processes rely on transferable evidence from adjacent fields (Wade et al., 2026). Exercise is therefore examined here as a candidate for research within a broader set of psychological, educational, and digital-health approaches, rather than as an established method of prevention or treatment.

Accordingly, this review complements prior general reviews by clarifying the conceptual boundary between problematic short-video use and the more strongly worded term “short-video addiction,” and by integrating short-video research, exercise psychology, digital-addiction research, and the I-PACE model within an evidence-informed four-pathway framework. Its contribution is not to assume that exercise is already effective, but to make a fragmented evidence base more testable: it distinguishes direct, reverse-direction, contextual, and transferable evidence and translates the proposed pathways into explicit priorities for temporal testing, outcome selection, intervention design, and implementation research.

## 2 Literature identification and narrative synthesis

### 2.1 Review design and scope

This theory-driven narrative review synthesized current evidence on exercise or physical activity in relation to problematic short-video use and examined potential psychological pathways relevant to future empirical and intervention research. Because direct evidence remains limited and methodologically heterogeneous, contextual short-video research and relevant literature from adjacent fields were also used to inform conceptual interpretation and framework development. The search was designed to support a structured, purposive narrative synthesis rather than to identify an exhaustive study universe for systematic review or quantitative pooling.

### 2.2 Information sources and search approach

China National Knowledge Infrastructure (CNKI), Web of Science, and PubMed were searched separately, with all searches completed on 24 March 2026. Chinese- and English-language publications were considered. The reference lists of relevant publications were also checked manually to identify additional literature pertinent to the conceptual, psychological, or intervention-oriented synthesis.

The searches were organized around three broad groups of concepts: short-video use, exercise or physical activity, and psychological processes or adjacent problematic digital-media behaviors. Synonymous and closely related terms within each group were combined with OR, whereas combinations across concept groups were constructed using AND. In CNKI, searches were conducted in topic, title, and keyword fields; Web of Science searches used the Topic field; and PubMed searches were conducted mainly in Title/Abstract fields. Search combinations were adapted to the structure and indexing of each database.

Representative short-video terms included “problematic short-video use,” “short video addiction,” “problematic short-form video use,” “short-video dependence,” and “short-video immersion,” together with their Chinese equivalents. Exercise-related terms included “exercise,” “physical activity,” “physical exercise,” and “exercise intervention.” Additional searches addressed self-control, emotion regulation, depression, anxiety, loneliness, social support, motivation, self-efficacy, resilience, internet addiction, digital addiction, behavioral addiction, problematic social media use, and the Interaction of Person–Affect–Cognition–Execution (I-PACE) model. The principal search terms, database fields, and database-adapted combinations are reported in Supplementary Table 1.

### 2.3 Publication selection and evidence organization

Titles and abstracts were assessed for relevance to the conceptual, psychological, and intervention questions addressed in the review. Full texts were examined when relevance could not be determined from the title or abstract alone. The first author conducted publication selection and evidence classification. The second author reviewed the final inclusion decisions and evidence organization, and questions concerning eligibility or classification were resolved through discussion between the two authors. Publications were retained when they directly or contextually informed the review of problematic short-video use or provided clearly transferable theoretical, methodological, or exercise-related evidence from adjacent fields. Publications were not retained when they were unrelated to problematic short-video use or adjacent problematic digital-media behaviors, addressed only general media exposure without a problematic-use indicator, or could not meaningfully contribute to the conceptual, psychological, exercise-related, or intervention-oriented synthesis.

A total of 48 publications were incorporated into the narrative synthesis. Each publication was assigned one primary evidential role according to its principal function in the review. Eight publications provided directly or closely relevant evidence on exercise or physical activity in relation to problematic short-video use or closely related short-video addiction indicators. Twenty-five provided contextual short-video evidence concerning conceptualization, measurement, platform and psychological mechanisms, consequences, or governance. Fifteen provided theoretical or transferable evidence relating to the I-PACE model, digital and behavioral addiction, exercise psychology and interventions, or digital behavior measurement. These categories were used to organize the narrative synthesis and were not treated as formal ratings of methodological quality or certainty of evidence. The literature-identification, relevance-assessment, and evidence-organization process is summarized in Supplementary Figure 1.

### 2.4 Narrative appraisal and synthesis

Interpretation considered study design, evidence direction, measurement approach, population and setting, and relevance to the review question. Greater caution was applied to findings based primarily on self-reported measures, inconsistent terminology or measurement of problematic short-video use, narrowly defined samples, or evidence derived from specific cultural and platform contexts.

Controlled intervention and longitudinal evidence, where available, received greater interpretive emphasis than cross-sectional associations. Cross-sectional mediation or moderation findings were used to identify plausible explanations but were not treated as evidence of temporal order or causal mechanisms. Reverse-direction studies were considered informative about possible reciprocal relationships, but they were not used to infer that exercise or physical activity reduces problematic short-video use. Evidence from adjacent fields informed theoretical interpretation and hypothesis development rather than being treated as direct evidence of short-video-specific effects.

The evidence was organized around four analytical pathways: reward substitution, emotional restoration, control restoration, and real-world reconnection. The type and directness of support for each pathway were considered separately, without assuming that the four pathways had equivalent empirical support. No formal risk-of-bias instrument or evidence-grading framework was applied. The eight publications directly or closely relevant to exercise or physical activity and problematic short-video use are summarized in Supplementary Table 2, including their designs, evidence directions, principal findings, and key interpretive limitations. The resulting framework was treated as a theory-informed synthesis intended to guide future testing, rather than as an established causal model.

## 3 Conceptual definition and analytical boundaries of problematic short-video use

### 3.1 Conceptual definition and core manifestations

Terminology in this field remains inconsistent. Studies use “short video addiction,” “problematic short-video use,” “short-video dependence,” “short-video immersion,” and “short-video overuse” for overlapping but non-equivalent phenomena (Dai et al., 2025; Li T. et al., 2025). This review uses problematic short-video use as an umbrella term for recurrent short-video engagement that is difficult to regulate and is associated with meaningful interference in daily functioning. “Short video addiction” is retained only when reporting the terminology, measures, or titles of original studies. The wording is descriptive rather than diagnostic: problematic short-video use is not recognized as a dedicated diagnosis in DSM-5-TR or ICD-11 (Dai et al., 2025; Li T. et al., 2025). Reported addiction-like features include impaired control, salience or preoccupation, mood-related use, conflict, and continued engagement despite adverse consequences, whereas evidence for tolerance remains limited.

Reported behavioral manifestations include automatic app opening, difficulty stopping, repeated return to the platform, and loss of time awareness (Dong et al., 2023). Short videos may also be used for boredom relief or emotional compensation, but these motives alone do not indicate problematic use (Wang et al., 2020; He, 2024). Functional relevance arises when the pattern displaces daily activities or interferes with learning, relationships, sleep, or psychological well-being (Song et al., 2025; Zhao and Lu, 2024). Song et al. (2025) organized these manifestations into substitution, dependence, and burden effects; this structure is useful for describing the phenomenon but should not be interpreted as a diagnostic system.

### 3.2 Conceptual boundaries and implications for exercise research

Problematic short-video use should be distinguished from intensive but well-regulated viewing and from broader constructs such as problematic internet or social-media use. Time spent alone is insufficient to identify problematic use; impaired control and interference with daily functioning are more informative. Short-video platforms also combine brief content, continuous swiping, personalized recommendation, rapid feedback, and low-threshold access, so findings from broader digital-use research cannot be transferred without qualification (Liao, 2024; Zhao and Lu, 2024). Qualitative research has further highlighted how platform affordances and algorithmic interaction can expand entertainment opportunities while leaving users with limited control over their engagement (Yan and Chen, 2023). Immersion, entertainment seeking, boredom relief, or procrastination may accompany short-video use, but none is interchangeable with problematic use in the absence of persistent dyscontrol or functional interference.

Patterns of short-video use may vary in degree, but current labels and measures do not establish a universal severity classification. Zhang et al. (2023), for example, assessed dependent use and excessive use as separate dimensions within a cross-national questionnaire model. This distinction is useful for examining how motivations, reliance on the platform, and adverse use patterns may be related, but it should not be treated as a validated sequence of clinical stages. These boundaries also affect the interpretation of exercise research. Less viewing time alone would not demonstrate reduced problematic short-video use unless it was accompanied by improved control or functioning. Likewise, changes in mood, self-control, or offline participation should not be interpreted as improvement in problematic use unless short-video behavior is assessed with a measure that captures impaired control and functional interference. Exercise-related findings should therefore be interpreted in terms of both regulation and functioning.

## 4 Potential psychological pathways linking exercise with problematic short-video use

This section examines four potential psychological pathways linking exercise with problematic short-video use: reward substitution, emotional restoration, control restoration, and real-world reconnection. They are used as overlapping analytical categories for comparing the available evidence rather than as independent causal mechanisms.

**Figure 1:**
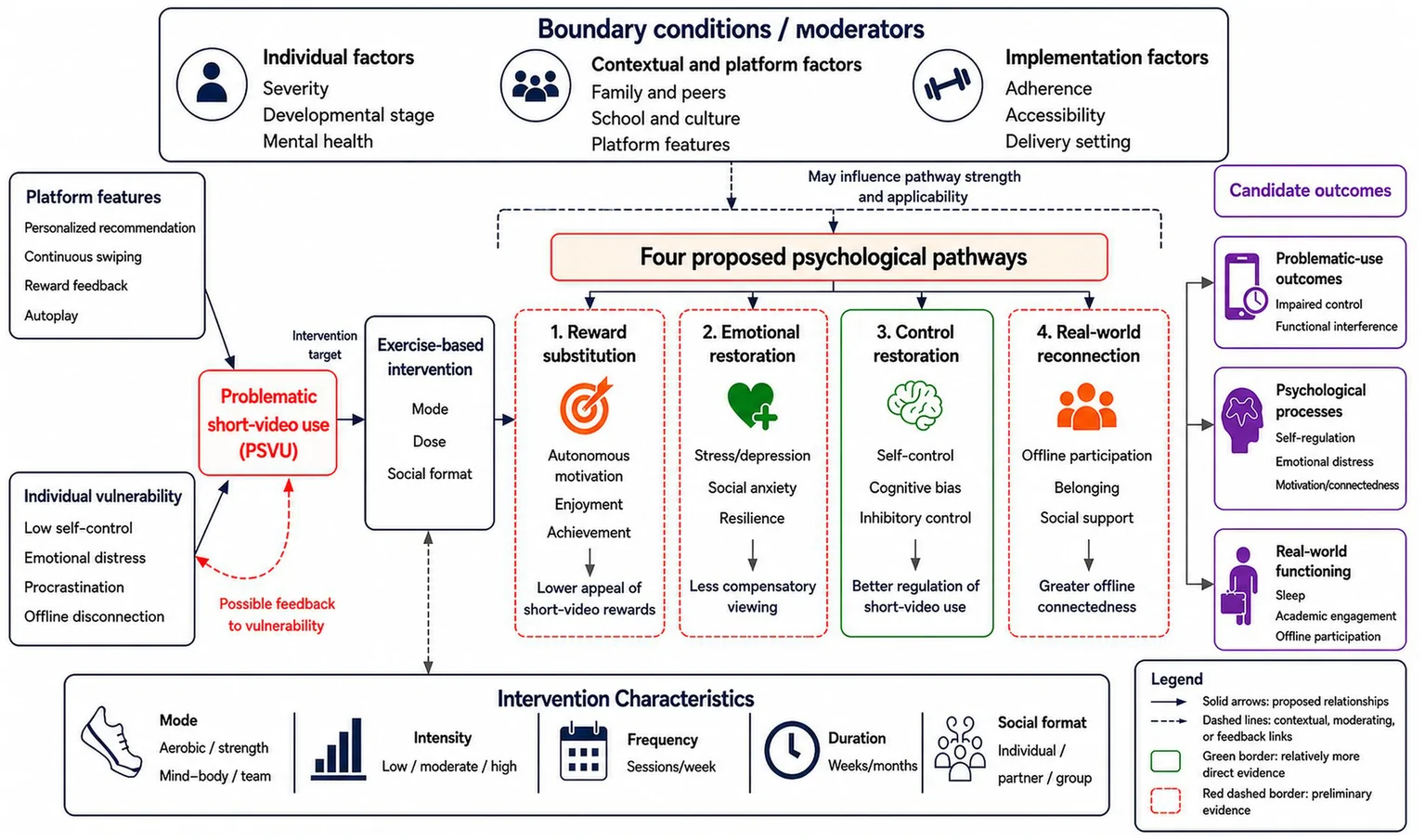
Proposed exercise-based framework for future testing in problematic short-video use. Exercise-based interventions are hypothesized to relate to problematic short-video use and associated functioning through four overlapping analytical pathways: reward substitution, emotional restoration, control restoration, and real-world reconnection. These pathways are not established causal mechanisms and may operate differently across individual, contextual, platform-related, and implementation conditions. The green solid border indicates relatively more direct intervention evidence, whereas red dashed borders indicate preliminary, association-based, reverse-direction, or transferable evidence. Dashed arrows represent proposed relationships requiring empirical testing.

Support differs across the four pathways. Control restoration has the clearest intervention relevance because it includes the only small controlled exercise study currently available. Emotional restoration is supported mainly by short-video-specific association and mediation studies, whereas reward substitution and real-world reconnection rely more heavily on cross-sectional, reverse-direction, conceptual, or transferable evidence. Figure 1 summarizes the proposed framework, and Table 1 compares the evidential role and principal limitations of each pathway. The order of presentation is conceptual rather than evidential.

**Table 1:** Evidence overview and narrative interpretation of the four proposed psychological pathways linking exercise with problematic short-video use.

Pathway	Candidate process	Representative evidence	Evidence type / limitations	Interpretation
Reward substitution	Exercise may offer alternative sources of satisfaction through enjoyment, skill development, achievement feedback, and task completion, potentially reducing the relative appeal of immediate swiping rewards.	Physical exercise was inversely associated with short-form video app addiction, with statistical indirect effects involving autonomous motivation and mental health literacy (Wu et al., 2026). Reverse-direction evidence linked higher short-video addiction with lower physical activity through lower self-efficacy and greater procrastination (Zhao and Kou, 2024).	Cross-sectional motivation-related and reverse-direction evidence only; no longitudinal or intervention test of the full sequence.	Hypothesis-generating pathway; exercise-related reward substitution has not been demonstrated.
Emotional restoration	Exercise may provide another context for mood regulation; whether exercise-related emotional change reduces compensatory short-video use remains untested.	Physical activity or exercise was associated with lower short-video addiction or problematic use, with statistical indirect effects involving social anxiety, depression, and psychological resilience (Wang et al., 2025; Yang et al., 2025; Peng et al., 2025). Yang et al. found context-specific rather than uniform associations.	Relatively more short-video-specific association evidence is available, but studies are mainly cross-sectional and self-reported; findings vary across use contexts.	One of the better-supported pathways, but current support remains associational and does not establish an exercise-mediated mechanism.
Control restoration	Structured exercise may provide opportunities to practice self-regulation involving self-control, cognitive bias, behavioral monitoring, and stopping control; transfer to short-video-use regulation remains untested.	A 16-week table-tennis intervention reported lower short-video-addiction and cognitive-bias scores and higher self-control (Wang et al., 2025). Cross-sectional and reverse-direction studies provide additional contextual evidence (Wang et al., 2025; He et al., 2024; Zhao and Kou, 2024).	One small controlled exercise study plus association-based evidence. The statistical indirect effect does not establish causal mediation or temporal ordering; replication with active controls and objective measures is needed.	Most intervention-relevant pathway, but direct support is limited to one study and should not be generalized.
**Real-world reconnection**	Socially structured exercise may create opportunities for offline participation, group belonging, and relational support; whether these changes reduce compensatory reliance on virtual connection remains untested.	Loneliness was associated with problematic short-video use (Mao and Jiang, 2023); social presence was associated with mobile short-video attachment (Jin and Liu, 2023); and review-level evidence summarized social support and family intimacy (Dai et al., 2025). Transferable youth-sport evidence concerned belonging and social support (Yao et al., 2025; Wade et al., 2026; Wei et al., 2025).	Short-video-specific evidence is cross-sectional, attachment-focused, or review-based. The exercise component is supported only indirectly; no study has tested the full exercise-mediated sequence.	Conceptually important and useful for hypothesis generation, but empirically under-tested and not established as an exercise-mediated pathway.

The four pathways are overlapping analytical categories rather than independent or equally validated causal mechanisms. Direct evidence refers to studies examining exercise or physical activity in relation to short-video-related outcomes; reverse-direction evidence refers to studies examining associations from problematic short-video use toward physical activity or related self-regulatory variables; transferable evidence derives from adjacent digital-addiction, exercise-psychology, sport, and physical-education research. The interpretation is narrative and does not represent formal evidence grading.

### 4.1 Reward and motivation: reward substitution

Short-video platforms can offer frequent opportunities for novelty and brief gratification through short-duration content, continuous swiping, rapid switching, personalized recommendation, and low-threshold access (Liao, 2024). Personalized recommendation may strengthen flow and immersive viewing, with self-control potentially moderating this association (Zhang et al., 2026). Motivation-related evidence also links escape, self-compensation, and stress relief with problematic short-video use, although these associations do not establish a single underlying reward mechanism (Chen et al., 2024).

The reward-substitution hypothesis proposes that exercise could provide alternative sources of satisfaction through enjoyment, skill development, achievement feedback, and task completion. It concerns a possible shift in the relative value of reward sources, rather than the simple replacement of short-video viewing time with exercise. No study has directly tested whether these exercise-related experiences reduce the appeal of short-video rewards and subsequently reduce problematic use.

Wu et al. (2026) reported an inverse association between physical exercise and short-form video app addiction among adolescents, with autonomous motivation and mental health literacy showing independent and sequential statistical indirect effects. The inclusion of autonomous motivation makes the study relevant to the reward-substitution hypothesis, but its cross-sectional design cannot establish whether exercise preceded motivational differences or whether adolescents with stronger motivation and psychological resources were more likely to exercise and report less problematic use.

Zhao and Kou (2024) examined the reverse direction. Higher short-video addiction was associated with lower physical activity, both directly and through lower self-efficacy and greater procrastination. This finding does not show that exercise reduces problematic short-video use; it instead suggests that problematic use, participation in real-world activity, and related psychological resources may be reciprocally associated.

Reward substitution therefore remains an untested exercise-mediated hypothesis rather than an established mechanism. Its most direct empirical test would examine whether increases in exercise enjoyment, perceived achievement, autonomy, and alternative real-world rewards precede reductions in the relative appeal and problematic use of short videos.

### 4.2 Emotion regulation: emotional restoration

Individuals may turn to short videos for brief emotional relief as well as for entertainment or information. Stress relief and escape from reality are frequently reported motives, and conceptual work suggests that psychological compensation may become more salient when emotional or relational needs are not adequately met offline (Wang et al., 2020; Xie and Jia, 2021). These motives alone do not indicate problematic use, but persistent reliance on short videos for coping may be relevant when use becomes difficult to regulate or interferes with daily functioning.

Exercise may provide another context for mood regulation through bodily activity, task completion, and, in some settings, social interaction. Experiences of accomplishment or support may be relevant to the emotional-restoration hypothesis, but no intervention study has directly tested whether exercise-related emotional change reduces compensatory short-video use.

Wang G. et al. (2025) reported that higher physical activity was associated with lower short-video addiction among college students, with self-control and social anxiety showing independent and sequential statistical indirect effects. These findings are relevant to both control restoration and emotional restoration. However, the cross-sectional questionnaire design cannot establish whether physical activity reduced social anxiety or whether social anxiety changed before problematic short-video use.

Yang et al. (2025) found that the associations varied across use contexts. Physical activity was directly associated only with lower nighttime use, while statistical indirect associations through depression were reported for nighttime, study-time, and leisure-time use and for short-video addiction. No significant total associations were found for daytime or overall use, and the observed effects were small. The results therefore do not support a uniform association between physical activity and all forms of short-video exposure.

In an adolescent sample, Peng et al. (2025) reported that physical exercise was inversely associated with depression and short-video addiction and positively associated with psychological resilience. Their model included statistical indirect effects through resilience and depression, including a sequential pathway. Because all variables were measured at the same time, the temporal order of these relationships remains unknown.

Emotional restoration has relatively more short-video-specific evidence than reward substitution or real-world reconnection, particularly for social anxiety, depression, and psychological resilience. Support nevertheless remains associational, varies across use contexts, and does not yet demonstrate that exercise-related emotional change reduces problematic short-video use. Its most direct empirical test would use repeated assessments to determine whether exercise-related changes in depression, social anxiety, or psychological resilience precede subsequent changes in problematic short-video use.

### 4.3 Executive control and self-regulation: control restoration

Research distinguishes goal-directed, instrumental short-video use from more ritualized engagement tied to habitual routines or particular contexts (Dong et al., 2023). The latter may be relevant to problematic short-video use when engagement becomes increasingly automatic and difficult to stop. Personalized recommendation, low-effort swiping, high stimulus density, and frequent feedback may also place demands on attention allocation, behavioral stopping, and self-monitoring, although the available evidence does not establish that these platform features cause impaired control. Subjective emptiness has been associated with more frequent scrolling (He, 2024), while other research has linked self-control with problematic short-form video use through automaticity and value-driven attention (Zhu and Fong, 2025). These findings make active monitoring and stopping control central candidate processes in this pathway.

Structured exercise often requires participants to follow goals and rules, sustain attention, regulate pace, inhibit immediate responses, and persist with a task. These features make exercise a plausible context in which to investigate self-regulatory practice, but transfer from exercise-related regulation to control over short-video use cannot be assumed.

The only controlled exercise study currently available was conducted by Wang K. et al. (2025). Sixty college students with elevated short-video-use or addiction indicators took part in a 16-week table-tennis program or maintained their usual activities. The intervention included three 60-min moderate-intensity sessions per week. After the program, the table-tennis group had lower short-video-addiction and cognitive-bias scores and higher self-control than the control group. The authors also reported a statistical indirect pathway involving cognitive bias and self-control. However, the reported mediation decomposition was not fully internally consistent, and the statistical indirect effect does not by itself demonstrate causal mediation or temporal ordering. This study therefore provides the clearest direct intervention evidence relevant to control restoration, but it does not establish the proposed mechanism conclusively. The study involved 60 students from one university, evaluated one exercise mode, used a usual-activity rather than an active exercise control, relied largely on self-reported central outcomes, and included no follow-up. Its findings therefore cannot be generalized to other exercise modes, populations, platforms, or longer-term outcomes.

Cross-sectional studies provide additional association-based evidence. Wang G. et al. (2025) reported an inverse association between physical activity and short-video addiction, with statistical indirect effects through self-control and social anxiety. He et al. (2024) similarly found that self-control partly accounted for the association between physical exercise and problematic TikTok use among adolescents, with parts of the model varying according to cumulative ecological risk. Neither study assigned exercise or established the direction of these relationships.

Other studies clarify the broader relevance of self-regulation without providing direct exercise evidence. Zhao and Kou (2024) found that higher short-video addiction was associated with lower physical activity through lower self-efficacy and greater procrastination, supporting a possible reciprocal relationship rather than an exercise effect. In a one-year longitudinal study, Liu et al. (2025) reported that problematic TikTok use predicted lower subsequent self-control, which was related to school engagement; the study did not test exercise.

Control restoration therefore has the clearest intervention relevance of the four pathways, but direct support still rests on one small controlled study. The clearest next step is replication in adequately powered trials with more diverse samples, active comparators, repeated assessments, follow-up, and objective or task-based measures to determine whether exercise-related changes in self-regulation precede changes in problematic short-video use.

### 4.4 Social relationships and real-world connectedness: real-world reconnection

Problematic short-video use has been associated with relational factors, although the evidence comes from different research traditions. In a cross-sectional survey of 1,277 middle-school students, loneliness was positively associated with problematic short-video use and statistically mediated the association between extraversion and problematic use (Mao and Jiang, 2023). Because all variables were measured at the same time, the study cannot establish whether loneliness preceded problematic use. A Bayesian network study of 2,548 adolescents likewise identified connections among parent–child technoference, parental psychological distance, loneliness, and short-video addiction risk, but its cross-sectional design did not establish temporal or causal direction (Shi et al., 2026). A recent review of research among college students also summarized inverse associations of perceived social support and family intimacy with short-video addiction (Dai et al., 2025).

Related research has examined attachment rather than problematic use. Jin and Liu (2023) found that social presence—comprising psychological involvement, co-presence, and perceived intimacy—was positively associated with adolescents’ mobile short-video attachment, with emotional responses showing a statistical indirect effect. Interview material also indicated that live-streaming and interactive functions could create experiences of participation, resonance, and perceived presence. However, mobile short-video attachment should not be treated as equivalent to problematic short-video use.

Conceptual analyses have further discussed unmet offline relational needs and compensatory or immersive short-video use (Xie and Jia, 2021; Wang, 2025). These propositions have not been tested as exercise-mediated pathways.

The relational relevance of exercise lies in the opportunities it may create for shared participation and social support. Adjacent youth-sport research provides indirect support for this possibility. In a systematic review of 189 studies, Wade et al. (2026) reported associations between sport participation and belonging, interpersonal skills, social support, and prosocial behavior, with team sports generally showing stronger and more consistent social benefits than individual sports. The review nevertheless concerned children and adolescents rather than individuals with problematic short-video use, and much of the included evidence was observational.

Other evidence is more preliminary. The Sport Education Model uses stable teams, role allocation, cooperation, and shared responsibility, which may create opportunities for teamwork and social interaction (Yao et al., 2025). However, the meta-analysis primarily evaluated broad physical-education learning outcomes, showed substantial heterogeneity, and provided sparse, very-low-certainty evidence for non-cognitive outcomes. Wei et al. (2025) also reported associations involving sport type, social support, and psychological resilience among Chinese adolescents, but its cross-sectional self-report design does not establish that team sports increased social support. Team sports, cooperative exercise, and stable-team physical-education formats may therefore be reasonable candidates for future testing because they provide repeated opportunities for interaction; they should not be interpreted as established or optimal exercise approaches for problematic short-video use.

Real-world reconnection overlaps with, but is distinct from, emotional restoration. Emotional restoration concerns changes in negative affect, including stress, depression, and social anxiety, whereas real-world reconnection concerns actual social participation, belonging, and relational support. The categories may interact: social support may reduce loneliness or social anxiety, while better emotional functioning may facilitate participation in real-world activities. They remain useful for organizing different aspects of the evidence, but they should not be interpreted as independent causal mechanisms.

Short-video-specific evidence supports the relevance of relational factors, but no study has tested whether socially structured exercise changes offline participation, belonging, or support and thereby reduces problematic short-video use. Its most direct empirical test would compare exercise formats that differ in social structure and examine whether changes in belonging, social support, and offline participation precede changes in problematic short-video use.

### 4.5 Integration of the proposed pathways and evidence interpretation

The four pathways provide a theory-driven structure for organizing potentially interacting psychological processes. Problematic short-video use may reflect the combined influence of reward seeking, emotion regulation, self-regulatory difficulty, social-environmental conditions, and platform-related features (Xiong et al., 2024). This interpretation is broadly consistent with the Interaction of Person–Affect–Cognition–Execution model, which emphasizes interactions among person-related vulnerabilities, affective and cognitive responses, executive control, and situational context (Brand et al., 2016, 2019). In this review, I-PACE serves as the principal organizing background; the four pathways are review-derived analytical categories rather than separate theories or co-equal causal mechanisms.

Within the I-PACE framework, reward substitution is most closely related to motivation and reward expectancy; emotional restoration to affective responses and coping; control restoration to cognitive bias and executive or inhibitory control; and real-world reconnection to social needs and person–context interaction. These categories overlap in practice. Social support may relate to loneliness or social anxiety, while emotional functioning may influence participation in offline activities. Figure 1 therefore presents potentially interacting processes rather than mutually exclusive channels or a confirmed synergy model.

Evidence differs across the pathways in both directness and research design. Control restoration has the clearest intervention relevance because one small controlled exercise study reported changes in problematic short-video use, cognitive bias, and self-control (Wang K. et al., 2025). Cross-sectional, reverse-direction, and non-exercise longitudinal findings provide additional context but do not demonstrate that exercise produces changes in self-regulation (Wang G. et al., 2025; He et al., 2024; Zhao and Kou, 2024; Liu et al., 2025). Emotional restoration has relatively more short-video-specific association evidence involving social anxiety, depression, and psychological resilience, although the findings are mainly cross-sectional and vary across use contexts (Wang G. et al., 2025; Yang et al., 2025; Peng et al., 2025).

Reward substitution relies mainly on cross-sectional motivation-related and reverse-direction evidence, and the proposed sequence from exercise to more rewarding real-world experiences and subsequently lower problematic short-video use remains untested (Wu et al., 2026; Zhao and Kou, 2024). Real-world reconnection is supported by loneliness-related evidence, research on mobile short-video attachment, review-level relational findings, and transferable youth-sport evidence; however, no study has tested the proposed exercise-mediated sequence (Mao and Jiang, 2023; Jin and Liu, 2023; Dai et al., 2025; Yao et al., 2025; Wade et al., 2026; Wei et al., 2025).

The relevance of the pathways may also vary across individual and contextual conditions. A grounded-theory study of short-video users aged 14–25 proposed exercise as a moderator within a broader model of psychological, content, environmental, and individual influences, although the model has not been quantitatively validated (Zhang, 2022). He et al. (2024), for example, found that cumulative ecological risk moderated parts of the cross-sectional association among physical exercise, self-control, and problematic TikTok use. This finding supports attention to developmental stage and family, peer, and school context, but it should not be interpreted as evidence that intervention effects differ across these conditions. The broader individual, contextual, and intervention-related moderators presented in Figure 1 should likewise be treated as hypotheses for future testing.

Taken together, the framework distinguishes where support is more direct, where it remains primarily contextual or transferable, and which hypotheses warrant priority in longitudinal or controlled testing. Table 1 summarizes the evidential role and principal limitations of each pathway; it does not represent a formal grading of evidence quality.

## 5 Implications for future exercise-based intervention research and support

Although current evidence does not support standard exercise prescriptions or validated pathway-specific matching, the four-pathway framework identifies four priorities for future intervention research: defining target populations, selecting theoretically relevant program features, assessing behavioral, psychological, and functional outcomes, and evaluating feasibility across implementation settings. These priorities are intended to guide intervention development and testing rather than serve as established recommendations for practice.

### 5.1 Considerations for identifying potential intervention targets

Evidence to date comes mainly from adolescents and college students, whose developmental and educational contexts differ. Within each population, users also vary in problem severity, psychological characteristics, and environmental conditions, so a single exercise program is unlikely to be appropriate for all users.

Relevant considerations may include the severity of problematic use, associated functional difficulties, psychological characteristics, and social context. Sleep, learning, interpersonal, or broader health problems may indicate that exercise alone would be insufficient (Dai et al., 2025). Self-control, emotional distress, and psychological resilience may also be relevant, but current studies have not validated these variables as criteria for selecting participants or assigning exercise programs (Wang G. et al., 2025; Peng et al., 2025).

Exercise is therefore better viewed as one possible component of broader behavioral support than as a stand-alone response. It may provide a structured alternative activity for some users, whereas marked loss of control, distress, or functional impairment may require additional psychological or contextual support. Current evidence does not provide validated criteria for determining who is likely to benefit from exercise alone.

### 5.2 Exercise-program design and evidence boundaries

Direct intervention evidence is limited to one small study. Wang K. et al. (2025) randomly assigned 60 college students from one university to a 16-week table-tennis program or a usual-activity control. The program involved three 60-min sessions per week at 60–80% of maximum heart rate. Relative to the control group, the table-tennis group showed lower short-video-addiction and cognitive-bias scores and higher self-control after the intervention. The single-site sample, reliance on self-reported central outcomes, absence of an active exercise comparator, and lack of follow-up limit generalizability and leave the specificity and durability of the findings uncertain.

Research on broader internet addiction provides only provisional, non-short-video-specific guidance. Deng (2014) evaluated a 10-week moderate-intensity program delivered three times per week, but a single study cannot establish a dose recommendation. A meta-analysis of nine studies reported lower internet-addiction scores following exercise, although heterogeneity remained high and subgroup analyses did not identify reliable differences by exercise type, duration, frequency, or intensity (Wang L. et al., 2025). A network meta-analysis ranked combined exercise highest, but all 15 included studies were conducted in China and the evidence was highly heterogeneous (Li Y. et al., 2025). These findings do not establish an optimal mode or dose for problematic short-video use.

The four-pathway framework may help generate hypotheses about relevant program features, but it should not be used to match individual pathways or users to particular exercise modes. Enjoyment, achievable challenge, a regular and manageable structure, opportunities to practice self-regulation, and social interaction are plausible features for future testing. Because the pathways overlap, the same feature may relate to several processes, and pathway-specific effects have not been compared directly.

Transferable executive-function evidence also requires caution. A dose–response network meta-analysis in children and adolescents reported small overall effects and an inverted-U pattern, with larger estimates for aerobic and skill/coordination activities at moderate doses (Li J. et al., 2025). Another meta-analysis reported improvements in inhibitory control, working memory, and cognitive flexibility following open-skill exercise, although heterogeneity was high and the apparent advantage of strategic activities was mainly limited to inhibitory control (Hu et al., 2025). These findings may inform hypotheses but do not identify an optimal exercise type or dose for problematic short-video use.

Aerobic exercise, resistance training, mindfulness-based or mind–body exercise, team sports, and skill-based activities are reasonable candidate modes for comparison, but none has established superiority. Aerobic exercise may be relatively straightforward to monitor; resistance training allows progressive and measurable tasks; mind–body exercise combines movement with attention and relaxation; and team or cooperative activities provide repeated opportunities for social interaction but require group access, scheduling, and willingness to participate socially (Zhou et al., 2025; Wade et al., 2026; Wei et al., 2025). Exercise selection should therefore be guided by participant preference, baseline capacity, safety, feasibility, and likely adherence rather than presumed mechanistic superiority.

Future trials need to specify exercise mode, intensity, session duration, weekly frequency, and total program length. Reporting attendance, adherence, dropout, adverse events, and continued participation after supervised delivery would help distinguish short-term effects from feasibility and maintenance.

### 5.3 Outcome assessment in exercise trials

A central methodological implication of the framework is a three-domain outcome structure encompassing short-video-specific problematic-use outcomes, candidate psychological processes, and real-world functioning.

Daily viewing time alone is insufficient as an outcome in exercise trials targeting problematic short-video use. A defensible minimum is a short-video-specific measure that captures impaired control and functional interference. Viewing duration and frequency may provide complementary information, but reduced exposure should not be interpreted as improvement without evidence of better regulation or functioning.

Behavioral assessment may also distinguish overall exposure from the timing and context of use. Relevant outcomes include nighttime or study-time viewing, difficulty stopping, and short-video-specific problematic-use scores (Yang et al., 2025; Wang K. et al., 2025). Because the cross-sectional findings of Yang et al. varied across use contexts, recording when and under what circumstances viewing occurs may be informative. Those findings do not demonstrate that exercise preferentially changes any particular form of short-video use.

Psychological variables should be prespecified and distinguished from primary behavioral outcomes. Self-control and cognitive bias currently have the most direct intervention relevance because both changed in the controlled table-tennis study (Wang K. et al., 2025). Evidence concerning autonomous motivation, mental health literacy, social anxiety, depression, and psychological resilience is mainly cross-sectional (Wu et al., 2026; Wang G. et al., 2025; Peng et al., 2025). Changes in these variables are therefore better treated as candidate explanatory processes or secondary outcomes unless temporal ordering and indirect effects are examined using repeated measurements.

Functional outcomes should reflect domains that were impaired or relevant at baseline. Depending on the population and research aim, these may include sleep quality or timing, school or academic engagement, and social or offline participation (Zhang et al., 2022; Liu et al., 2025). Such outcomes help determine whether behavioral change has practical relevance, although current evidence does not establish that reductions in problematic use following exercise necessarily improve functioning.

Measures should match the construct under study. General internet-, smartphone-, or social-media-addiction scales are not interchangeable with instruments developed specifically for short-video use. The Short Video Addiction Behavior Scale includes substitution, dependence, and burden domains, describing displacement of offline activities, preferential engagement and psychological dependence, and reported harms, respectively (Song et al., 2025). Domain scores may help identify which aspects of problematic use changed, but they do not reveal the psychological process through which exercise acted. Further validation is also required across age groups, cultures, platforms, and intervention settings.

Questionnaires may be complemented by device-recorded use logs, ecological momentary assessment, or passive sensing, depending on whether the aim is to assess exposure, momentary context, or patterns of device use (Parry et al., 2021; Ryding and Kuss, 2020; Ahn et al., 2025). Assessment at baseline, during intervention, immediately afterward, and at follow-up would provide a stronger basis for examining the temporal ordering, durability, and maintenance of change.

### 5.4 Implementation settings and feasibility

Implementation will depend on the educational, family, and platform contexts in which participants live, study, and exercise. Direct evidence is currently limited to one small university-based trial, and no study has compared the feasibility of exercise delivery across settings. Potential implementation routes should therefore be treated as options for evaluation rather than established models.

For adolescents, physical education classes and extracurricular activities may provide structured delivery opportunities. Feasibility would nevertheless depend on timetable space, qualified staff, facilities, supervision, safety procedures, and access to psychological support for students with substantial distress or functional impairment. Programs embedded in existing classes may also differ from voluntary extracurricular programs in reach, acceptability, and participant motivation.

For college students, elective courses, sports clubs, and campus recreation may be more plausible routes. The university-based table-tennis study does not establish feasibility in institutions with different facilities, staffing, schedules, or student populations (Wang K. et al., 2025). Academic demands, access to exercise spaces, program costs, and willingness to participate may affect recruitment and continued involvement. The added value of combining exercise with media-literacy or psychological components has not been tested.

Family involvement may be relevant mainly to adolescent programs, but it should not be assumed to be uniformly beneficial or feasible. Commentaries have proposed shared activity, screen-free periods, and face-to-face alternatives (Wang, 2025; Su and Song, 2024), yet these components have not been evaluated in exercise trials. Cross-sectional findings concerning cumulative ecological risk support measuring family, peer, and school context but do not establish how these factors influence intervention outcomes (He et al., 2024). The appropriate level of family involvement may vary according to age, relationships, participant preference, available time, and support needs.

Exercise also does not remove platform-level cues such as personalized recommendation, continuous swiping, or autoplay (Liao, 2024; Zhao and Lu, 2024). Whether exercise provides additional benefit when combined with time reminders, platform-based protection settings, media-literacy education, or psychological support remains unknown. Broader digital-addiction research has similarly located prevention and intervention across individual, technological, and governance levels (Liang et al., 2026). Exercise-based programs should therefore not be framed as substitutes for platform-level or broader behavioral approaches.

Feasibility studies would be strengthened by reporting recruitment, reach, acceptability, delivery fidelity, staffing and facility requirements, resource use, and reasons for non-participation. Such information is needed before exercise-based support can be judged scalable across schools, universities, or community settings.

## 6 Discussion

This review organizes fragmented evidence on exercise and problematic short-video use within an evidence-informed, multi-pathway framework. By distinguishing four overlapping potential pathways and comparing the directness and design of their supporting evidence, it provides a structured agenda for future intervention research. Support nevertheless remains uneven: control restoration is the only pathway with direct intervention evidence, whereas emotional restoration is supported mainly by short-video-specific associations and reward substitution and real-world reconnection rely more heavily on indirect or transferable findings. The framework should therefore be interpreted as a research agenda rather than a validated causal model or exercise prescription.

This was a theory-driven narrative review. Literature identification was purposive rather than exhaustive, and no formal risk-of-bias assessment or quantitative synthesis was undertaken. Pooled effects and the overall efficacy of exercise cannot be estimated. Publication bias could not be assessed formally, and positive findings may be overrepresented. The conclusions should therefore be read as a critical interpretation of current evidence rather than an estimate of intervention effectiveness.

### 6.1 Terminological and measurement inconsistencies

Terminology remains inconsistent across the literature. Labels such as “short-video addiction,” “problematic short-video use,” “dependence,” “immersion,” and “overuse” are often used without clear operational distinctions, making it difficult to separate intensive but well-regulated viewing from more persistent patterns involving impaired control or functional interference (Dai et al., 2025). This review therefore uses problematic short-video use as the broader research term and retains “short-video addiction” only when reporting the terminology of an original study or measure, without treating it as a formally recognized clinical diagnosis.

Measurement differences create a further problem. General digital-addiction scales and short-video-specific instruments do not necessarily assess the same construct, and even short-video-specific measures require further validation across age groups, cultures, platforms, and intervention settings. Their total or domain scores may describe reported problem features, but they do not identify the psychological process through which exercise acted (Song et al., 2025).

Several directly relevant studies assessed physical activity or exercise, psychological variables, and problematic short-video use using concurrent self-report questionnaires (He et al., 2024; Wu et al., 2026; Wang G. et al., 2025; Peng et al., 2025). Such designs are vulnerable to recall and social-desirability bias as well as common-method variance, which may inflate associations among variables reported by the same participant at the same time. Cross-sectional statistical indirect effects are especially difficult to interpret under these conditions because shared measurement method and uncertain temporal ordering cannot be separated from the proposed psychological pathway.

Self-reported physical activity is also not equivalent to a verified structured exercise dose, and reported viewing duration may differ from device-recorded use. Short-video-specific questionnaires could therefore be complemented, where feasible, by platform-use logs, ecological momentary assessment, or passive sensing (Parry et al., 2021; Ryding and Kuss, 2020; Ahn et al., 2025). These measures may improve the precision of exposure assessment, but they cannot replace the assessment of impaired control and functional interference that defines problematic use.

### 6.2 Causal inference and intervention design

Evidence directly linking physical activity or exercise with problematic short-video use is mixed rather than consistently protective. Several cross-sectional studies reported inverse associations and statistical indirect effects involving self-control, social anxiety, depression, psychological resilience, or autonomous motivation (Wu et al., 2026; Wang G. et al., 2025; Peng et al., 2025; He et al., 2024). Yang et al. (2025), however, found a direct association only with nighttime use, no significant total associations for daytime or overall use, and generally small effects. Associations may therefore depend on the form and context of short-video use rather than reflecting a uniform relationship.

Directionality remains unresolved. Cross-sectional models cannot determine whether greater physical activity precedes lower problematic short-video use, whether problematic use contributes to withdrawal from physical activity, or whether both are related to other psychological or contextual factors. Zhao and Kou (2024) examined the reverse direction and found that higher short-video addiction was associated with lower physical activity, both directly and through lower self-efficacy and greater procrastination. Taken together, the available studies are more consistent with potentially reciprocal and context-dependent relationships than with a demonstrated protective effect of physical activity.

The only controlled exercise study reported lower short-video-addiction and cognitive-bias scores and higher self-control after a 16-week table-tennis program (Wang K. et al., 2025). This study provides the most direct intervention evidence currently available, but it does not by itself establish efficacy or mechanism. The single-university sample, usual-activity rather than active-exercise control, reliance largely on self-reported central outcomes, and absence of follow-up limit causal interpretation, specificity, generalizability, and durability. The reported statistical indirect pathway is consistent with the control-restoration hypothesis but does not demonstrate causal mediation or temporal ordering.

Resolving these questions will require adequately powered randomized trials with short-video-specific primary outcomes and comparators suited to the research question. Active-exercise controls are particularly relevant when testing whether findings are specific to an exercise modality rather than participation in a structured activity more generally. Repeated assessments can clarify temporal ordering and durability, while reporting adherence, dropout, adverse events, and continued participation would help distinguish short-term changes from feasible and sustained effects. Current evidence does not justify assigning particular forms of exercise to the four proposed pathways.

### 6.3 Testing candidate psychological processes

Research has examined self-control, cognitive bias, social anxiety, depression, psychological resilience, autonomous motivation, and mental health literacy as possible explanatory variables. Most evidence comes from cross-sectional mediation or moderation models (Wu et al., 2026; Wang G. et al., 2025; Peng et al., 2025), whereas the only controlled exercise study assessed changes in cognitive bias and self-control (Wang K. et al., 2025). These findings identify variables that warrant further examination, but they do not establish that those variables mediate an exercise effect. Statistical indirect effects based on variables measured at the same time cannot determine temporal ordering, and sequential paths should not be treated as evidence of a sequence of psychological changes.

Testing these processes also requires a clearer distinction between mediation and moderation. Self-control or cognitive bias may be examined as possible mediators, whereas developmental stage, baseline severity, cumulative ecological risk, or family and school context may be more appropriately examined as moderators. Because the four pathways overlap, individual variables need not belong exclusively to one pathway. Including several variables in the same statistical model does not, by itself, validate an integrated mechanism.

Repeated measurement is necessary to determine whether change in a proposed process precedes later change in problematic short-video use. Structural equation modeling can compare prespecified models or estimate indirect paths, but satisfactory fit cannot establish causality or exclude reverse direction. Measurement would also be strengthened by combining validated questionnaires, where feasible, with task-based measures of self-control or attentional bias, device-recorded platform use, ecological momentary assessment, or passive sensing (Parry et al., 2021; Ryding and Kuss, 2020; Ahn et al., 2025). Exercise exposure and digital-use outcomes should be documented separately, because no single measure identifies the process through which exercise may influence problematic use.

### 6.4 Application boundaries and generalizability

Application boundaries remain uncertain because existing studies have not compared intervention outcomes across levels of problematic-use severity or tested whether psychological distress, baseline physical activity, or social context modifies response. Current evidence cannot support severity-based assignment of exercise or identify a subgroup for whom exercise alone would be sufficient. The cross-sectional finding that cumulative ecological risk moderated parts of the associations among physical exercise, self-control, and problematic TikTok use does not resolve these questions because no intervention was tested (He et al., 2024). Family, peer, and school conditions are therefore better treated as possible contextual moderators than as established criteria for assigning an intervention.

Most of the evidence comes from Chinese adolescents and college students. Applicability to adults and older adults cannot be assumed because motives for short-video use, health status, functional capacity, digital habits, and the feasibility and safety of structured exercise may differ from those of student samples. Studies involving older populations would require age-appropriate measures, exercise protocols, settings, and safety procedures rather than direct transfer of student-based programs.

Cross-national research on excessive short-video-app use among college students has begun, but it does not show that exercise-related associations or intervention effects generalize across cultures (Zhang et al., 2023). Educational systems, family involvement, attitudes toward organized exercise, facility access, and platform governance may vary across countries. Proposals developed for Chinese adolescents should not therefore be treated as directly applicable to Western or other cultural settings without adaptation and empirical testing (Zhao and Zhao, 2025).

Platform differences create an additional boundary. Much of the literature concerns Douyin or TikTok, whereas Instagram Reels, YouTube Shorts, and other services may differ in recommendation systems, social functions, content ecology, user populations, and patterns of use. Cross-cultural and cross-platform research will need comparable age groups, measures that function similarly across languages and settings, clear identification of the platform studied, and documentation of local implementation conditions. Until such evidence is available, the applicability of the framework across populations and environments remains an empirical question.

## 7 Conclusion

This review synthesized current evidence on how exercise may relate to problematic short-video use through four overlapping pathways: control restoration, emotional restoration, reward substitution, and real-world reconnection. The available evidence suggests that exercise may be relevant to problematic short-video use, but support differs substantially across pathways and remains limited by the predominance of cross-sectional studies and the scarcity of controlled intervention evidence.

Among the four pathways, control restoration currently has the strongest direct support because it includes the only controlled exercise study targeting problematic short-video use. However, the available findings do not establish a causal mechanism, identify an optimal exercise approach, or support pathway-specific exercise prescriptions. Evidence for emotional restoration, reward substitution, and real-world reconnection remains primarily associative or based on related research domains.

Exercise should therefore be considered a potential component of broader support strategies rather than a standalone solution for problematic short-video use. Future research requires well-designed longitudinal and randomized studies that clarify temporal relationships, evaluate short-video-specific outcomes, examine potential psychological processes, and determine the feasibility and durability of exercise-based approaches across different populations and settings.

Together, these distinctions provide a structured basis for prioritizing testable pathways, selecting short-video-specific and functional outcomes, and designing longitudinal and controlled studies.
