# Supplementary material for: Exercise-based interventions for problematic short-video use: a narrative review of current evidence and potential psychological pathways
Source: Front Psychol. 2026 Sep 3;17:1865897. doi: 10.3389/fpsyg.2026.1865897 (PMC13581819; doi:10.3389/fpsyg.2026.1865897)
Supplement: Supplementary file 1 [file image_1.pdf]

Supplementary Figure 1

Narrative literature-identification and evidence-organization process

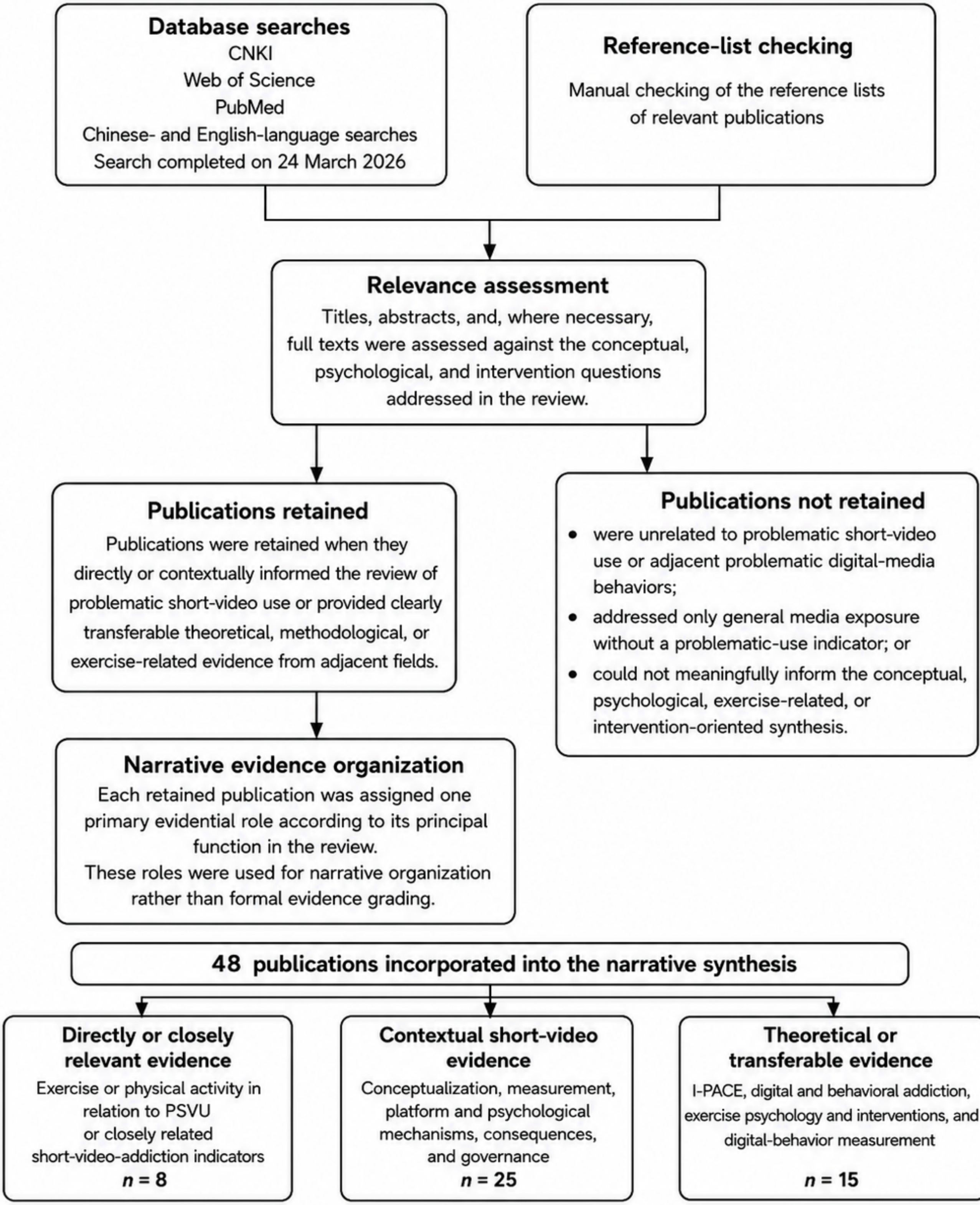

Note. This non-PRISMA diagram summarizes the purposive literature-identification, relevance-assessment, and evidence-organization process used in this theory-driven narrative review. The three categories indicate each publication's primary narrative role rather than its methodological quality or certainty of evidence.
