# Supplementary material for: Exercise-based interventions for problematic short-video use: a narrative review of current evidence and potential psychological pathways
Source: Front Psychol. 2026 Sep 3;17:1865897. doi: 10.3389/fpsyg.2026.1865897 (PMC13581819; doi:10.3389/fpsyg.2026.1865897)
Supplement: Supplementary file 2 [file table_1.docx]

**Supplementary Table 1. Principal search terms and database-adapted literature-identification notes**

Note. This table summarizes the principal search terms, database fields, and database-adapted combinations used in this theory-driven narrative review. It is not a complete saved search history, a PRISMA flow diagram, or a record of formal systematic screening.

| **Source / component** | **How literature was identified** | **Principal terms or coverage** | **Limits / date** | **Role in the review** |
| --- | --- | --- | --- | --- |
| CNKI | Keyword searches were conducted in topic, title, and keyword fields, using combinations relevant to the review questions. | 短视频成瘾; 问题性短视频使用; 短视频依赖; 短视频沉浸; 短视频过度使用; 体育锻炼; 身体活动; 运动干预; 自我控制; 情绪调节; 抑郁; 焦虑; 社会支持; 动机; 自我效能; 心理韧性. | Chinese-language publications; search completed on 24 March 2026. | To locate Chinese empirical, conceptual, and review publications on problematic short-video use, exercise or physical activity, and related psychological processes. |
| Web of Science | Keyword searches were conducted in Topic fields, with combinations adapted to the database indexing and the questions addressed in the review. | short video addiction; short-video addiction; problematic short-video use; problematic short-form video use; short-form video addiction; TikTok addiction; short-video dependence; short-video immersion; exercise; physical activity; sport; psychological-process terms. | English-language publications; search completed on 24 March 2026. | To identify international research on problematic short-video use and its links with exercise, physical activity, and relevant psychological processes. |
| PubMed | Keyword searches were conducted mainly in Title/Abstract fields. | Short-video terms combined with exercise, physical activity, physical exercise, exercise intervention, sport, self-control, emotion regulation, depression, anxiety, social support, motivation, self-efficacy, and resilience. | English-language publications; search completed on 24 March 2026. | To identify biomedical, psychological, and exercise-health literature relevant to the review questions. |
| Reference-list checking | Reference lists of relevant publications were checked manually to locate additional sources. | internet addiction; digital addiction; behavioral addiction; problematic social media use; smartphone addiction; exercise psychology; I-PACE model. | Chinese- and English-language sources; checking completed on 24 March 2026. | To identify additional conceptual, theoretical, and transferable evidence from adjacent fields. |
| Publication selection and organization | Publications were selected purposively according to their relevance to the conceptual, psychological, and intervention questions addressed in the review. | 48 publications were organized by one primary role: 8 directly or closely relevant publications, 25 contextual short-video publications, and 15 theoretical or transferable publications. | Purposive narrative selection; no numerical systematic-review screening flow was constructed. | To organize heterogeneous literature transparently without implying formal quality grading or certainty-of-evidence assessment. |
