# Supplementary material for: Exercise-based interventions for problematic short-video use: a narrative review of current evidence and potential psychological pathways
Source: Front Psychol. 2026 Sep 3;17:1865897. doi: 10.3389/fpsyg.2026.1865897 (PMC13581819; doi:10.3389/fpsyg.2026.1865897)
Supplement: Supplementary file 3 [file table_2.docx]

**Supplementary Table 2. Characteristics, evidence direction, and interpretive limitations of publications directly or closely relevant to exercise/physical activity and problematic short-video use**

**Note.** The eight publications comprise one controlled exercise-intervention study, five forward-direction cross-sectional association or mediation studies, one reverse-direction cross-sectional study, and one narrative review. “Directly or closely relevant” describes topical relevance, not equal evidential strength. Cross-sectional mediation findings are interpreted as associations and plausible hypotheses rather than causal mechanisms. The term “problematic short-video use” is used as the review-level umbrella term; original study terminology is retained where appropriate.

| **Study / article** | **Design and participants** | **Exercise / physical-activity component** | **Short-video outcome and candidate process** | **Main finding** | **Evidence role in this review and key interpretive cautions** |
| --- | --- | --- | --- | --- | --- |
| **Wang K. et al. (2025)** | Controlled pre-post intervention; 60 college students with high short-video use/addiction indicators from one university in Chengdu, China; randomly allocated to intervention (n = 30) or usual-activity control (n = 30). | Sixteen-week table-tennis program; three 60-min sessions per week; moderate intensity (60-80% HRmax). The control group maintained usual activities. | College Students Short Video Addiction Scale; negative cognitive-processing bias and self-control were tested as sequential mediators. | Compared with the control group, the table-tennis group showed lower short-video addiction and cognitive bias and higher self-control after the intervention. Cognitive bias and self-control formed a reported chain-mediation pathway. | Clearest direct intervention evidence, used mainly for the control-restoration pathway and secondarily for cognitive-bias interpretation. Promising but not definitive: small sample, single university, one exercise form, self-reported central outcomes, no active exercise-control group, and no follow-up. Results should not be generalized to all exercise modes, age groups, or platforms. In addition, the reported mediation decomposition was not arithmetically consistent as presented: the direct effect (β = −0.25) and total indirect effect (β = −0.41) did not sum to the reported total effect (β = −0.60), while the corresponding proportions (41.67% and 68.33%) summed to 110%. The source article did not explain this discrepancy; therefore, the mediation estimates were interpreted cautiously. |
| **He et al. (2024)** | Cross-sectional moderated-mediation study; 756 adolescents from Chongqing and Chengdu, China. | Physical-exercise level measured with the Physical Activity Rating Scale-3; no assigned intervention and no separation of specific exercise modes. | Problematic TikTok Use Scale; self-control was examined as a mediator and cumulative ecological risk as a moderator. | Physical exercise was negatively associated with short-video addiction (beta = -0.30). Self-control partially mediated the association, and cumulative ecological risk moderated parts of the model. | Forward-direction association evidence used mainly for control restoration and contextual boundary conditions. The cross-sectional design cannot establish temporal order or causality; the survey occurred near the end of the COVID-19 period, exercise type was not differentiated, and subgroup/generalizability questions remain. |
| **Wang G. et al. (2025)** | Cross-sectional questionnaire study; 650 college students recruited by convenience sampling in China. | Self-reported physical-activity level; no assigned intervention. | Short-video addiction; self-control and social anxiety were tested as independent and sequential mediators. | Higher physical activity was associated with lower short-video addiction. Self-control and social anxiety showed independent and chain-mediation patterns. | Association-based support for the control-restoration and emotional-restoration pathways; social-anxiety findings may also inform hypotheses about offline social functioning. Causal direction is uncertain because of the cross-sectional, convenience-sample design; reverse causality and unmeasured third variables cannot be excluded, and all central variables were questionnaire-based. |
| **Yang et al. (2025)** | Cross-sectional online survey and structural-equation modeling; 1,172 university students from five Chinese universities. | Physical activity assessed by reported frequency and duration rather than a validated multidimensional physical-activity instrument. | Nighttime, daytime, study-time, leisure-time, and overall short-video use, plus short-video addiction; depression was tested as a mediator. | Physical activity had a direct association only with lower nighttime use. Indirect associations through depression were found for nighttime, study-time, and leisure-time use and for short-video addiction. No significant total effects were found for daytime or overall use, and the observed effects were small. | Used for emotional restoration and to show that short-video behavior varies by context. Particularly important as mixed/limited evidence rather than uniformly positive support. Cross-sectional design, nonvalidated physical-activity measurement, online recruitment, gender imbalance, possible selection bias, small effects, and several null results restrict causal and practical claims. |
| **Peng et al. (2025)** | Cross-sectional chain-mediation study; 1,002 adolescents recruited from six primary and secondary schools in five Chinese provinces. | Physical-exercise level assessed by questionnaire; no assigned intervention. | Short-video addiction; psychological resilience and depression were examined as sequential mediators. | Physical exercise was negatively correlated with depression and short-video addiction and positively correlated with psychological resilience. Resilience and depression showed a sequential mediation pattern. | Forward-direction association evidence used mainly for the emotional-restoration pathway and psychological-resource interpretation. Convenience sampling, cross-sectional design, regional representativeness limits, and very brief measures of exercise and resilience restrict causal and measurement precision. |
| **Wu et al. (2026)** | Cross-sectional questionnaire study; 644 junior and senior high-school students from Shandong Province, China. | Physical-exercise level measured by questionnaire; no assigned intervention. | Short-form video app addiction; autonomous motivation and mental-health literacy were examined as independent and sequential mediators. | Physical exercise was negatively associated with short-form video app addiction. Autonomous motivation and mental-health literacy showed independent and chain-mediation patterns. | Used mainly for reward/motivation and psychological-resource hypotheses, including a cautious link to self-determination theory. The study explicitly cannot establish temporal sequencing; alternative reverse pathways are possible. A geographically homogeneous, volunteer, self-report sample and same-time measurement limit external validity and causal interpretation. |
| **Zhao and Kou (2024)** | Cross-sectional chain-mediation study; 304 undergraduates from two normal universities in Henan Province, China; stratified random selection by gender and grade. | Physical activity was the outcome variable, measured with the Physical Activity Rating Scale-3; it was not an intervention or antecedent exposure. | Short-video addiction was the predictor; self-efficacy and procrastination were examined as independent and sequential mediators. | Higher short-video addiction was associated with lower physical activity, directly and indirectly through lower self-efficacy and greater procrastination. | Reverse-direction evidence only. It supports the possibility of reciprocal relations but does not show that exercise reduces problematic short-video use. One-time questionnaire data cannot establish causality or temporal order, and the model tested only one directional specification with a limited set of mediators. |
| **Su and Song (2024)** | Chinese narrative literature-review article; no original participant sample or intervention dataset. | Physical exercise discussed as a possible non-pharmacological approach to prevention or alleviation of addictive behavior. | Adolescent short-video addiction and its physical, psychological, and social consequences; no original mediation or intervention analysis. | The article summarized prior literature, discussed possible roles of exercise, and proposed practical suggestions for adolescent prevention and intervention. | Contextual and practical background only. It is not direct empirical or intervention evidence and provides no original effect estimate or causal test. It should not be counted as confirmation of any of the four proposed pathways. |

**Abbreviations:** HRmax, maximum heart rate.
